# Maxillary Sinus Pneumatization Following Extractions in Riyadh, Saudi Arabia: A Cross-sectional Study

**DOI:** 10.7759/cureus.6611

**Published:** 2020-01-09

**Authors:** Sadeem Alqahtani, Ahmad Alsheraimi, Ahmad Alshareef, Rana Alsaban, Alwaleed Alqahtani, Mohammad Almgran, Malek Eldesouky, Ahmad Al-Omar

**Affiliations:** 1 Dentistry, King Saud University, Riyadh, SAU; 2 Radiology, Security Forces Hospital, Riyadh, SAU; 3 Oral and Maxillofacial Surgery, King Saud University, Riyadh, SAU

**Keywords:** maxillary sinus pneumatization, tooth extraction, dental implants, vertical height

## Abstract

Introduction

It is generally agreed that tooth extraction may lead to maxillary sinus pneumatization, resulting in a union between the sinus floor and the crest of the remaining bone in extreme cases. Studies that compared pre- and postextraction radiographs suggest that maxillary sinus pneumatization may occur after posterior tooth extractions. This study’s aim was to establish the prevalence of maxillary sinus pneumatization following extractions in Riyadh, Saudi Arabia.

Material and methodology

In this cross-sectional study, 282 panoramic images were randomly selected from the radiology department of the Dental University Hospital in Riyadh, Saudi Arabia, from the years 2015 to 2018. The radiographs included were of patients who had one of the following teeth extracted: the second premolar, the first molar, or the second molar. These radiographs were then evaluated for sinus pneumatization following extractions. The distance between the sinus floor and the inferior border of the alveolar ridge after the extraction was reviewed and assessed for all images.

Result

The six teeth that were assessed in this study were: 17, 16, 15, 25, 26, and 27. In relation to distribution (unilateral and bilateral), the results showed a statistically significant difference, especially for tooth numbers 16, 15, and 26. The mean values of change in sinus were significantly higher in the unilateral site than the bilateral site. However, the data did not provide any significant difference for the other three teeth (17, 25, and 27). The gender and molar side groups showed no statistical significance.

Conclusion

The results of the study showed that maxillary sinus pneumatization may occur after posterior tooth extraction.

## Introduction

The maxillary sinus, also known as the “antrum of Highmore,” is the largest of the paranasal sinuses and the first to develop embryologically at 16 weeks of gestation [1]. The sinus is pyramidal in shape and is composed of four walls, including a base formed by the lateral wall of the nose and an apex that extends into the zygomatic process [2-3]. It lies within the body of the maxillary bone on the lateral side of the nasal cavity [4].

Sinus pneumatization is a continuous physiological process that causes the paranasal sinuses to increase in volume [5]. Sinuses give resonance to voice, contribute to the shape of the face, and provide some degree of warmth and humidification to inspired air [6].

After birth, the maxillary and ethmoidal sinuses are fully developed [7] and continue to pneumatize as the permanent teeth erupt [8]. The pneumatization extends inferomedially, through nearby bony elements into the hard palate, laterally into the zygomatic bone, and posteriorly into the ethmoids [9]. According to some authors, the most common site to undergo pneumatization is the anteromedial wall of the maxillary sinus [10-11].

However, pneumatization may sometimes be extensive and may expose the roots, resulting in the engagement of the maxillary molar and premolar roots within the floor of the sinus. This may lead to complications during extractions and difficulties during implant placement [12-13].

It is believed that tooth loss induces maxillary sinus pneumatization, which may lead to a union between the sinus floor and the crest of the alveolar bone in extreme cases [14]. Some studies that compared pre- and post-extraction radiographs suggest that maxillary sinus pneumatization may occur after posterior tooth extraction [8]. A study conducted in Israel that compared the dimensional changes in the alveolar ridge and the corresponding maxillary sinus following tooth extraction, with or without socket preservation, showed that tooth extractions in the posterior maxilla may lead to sinus pneumatization and crestal bone loss [15].

This study aimed to assess maxillary sinus pneumatization following extractions.

## Materials and methods

A total of 282 panoramic radiographs in this retrospective study were selected randomly from archived dental records of patients treated in the Dental University Hospital at King Saud University in Riyadh, Saudi Arabia. They were all obtained with a Planmeca ProMax 3D Max machine (Helsinki, Finland). Digital panoramic radiographs were used to assess maxillary sinus pneumatization following tooth extractions.

Radiographs of patients who had a tooth extraction of either the second premolar, the first molar, or the second molar were included in the study. Images of patients with a history of sinus diseases, sinusitis, pathological lesions, or bone preservation procedures were excluded.

**Figure 1:**
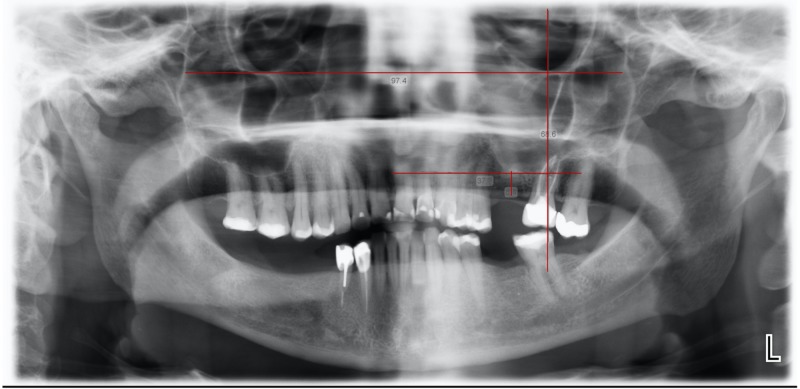
Panoramic image illustrating the reference lines drawn and the perpendicular distances measured in the study

The radiographs selected showed the roots of the posterior maxillary teeth, the maxillary sinus floor, and the inferior orbital margins. Three reference lines were marked on all radiographs under standard conditions (Figure 1): one line joining the most inferior points of both orbital margins, a vertical line passing through the orbit, bisecting it into two equal halves, and a third line running horizontally, parallel to the inferior border of the sinus.

Measurements taken included the distance between the maxillary sinus floor to either the second premolar, the first molar, or the second molar, as these are the teeth whose roots are closest to the sinus and crest of the alveolar ridge.

The data were analyzed using SPSS 24.0 statistical software (IBM Inc., Chicago, USA). Descriptive statistics, including mean, standard deviation, frequencies, and percentages, were used to describe the quantitative and categorical variables. Student’s t-test for independent samples was used to compare the mean values of change in the sinus floor position in relation to gender, site, and molar side. One-way analysis of variance was used to compare the mean values of change in sinus in relation to the age groups while Tukey’s multiple comparison test was used in relation to molar type. A p-value of <0.05 was used to report the statistical significance of results.

The teeth nomenclature selected and used in this paper was the World Dental Federation (FDI) two-digit numbering system for the purpose of identifying teeth.

## Results

A total of 282 radiographs were selected and analyzed. Out of the 282 study subjects, 158 subjects (56%) were females and 147 subjects (52.1%) were between the ages of 31 and 50 years. The distribution was unilateral in 171 subjects (60.6%) of the subjects and bilateral in 111 subjects (39.4%). The six teeth evaluated in this study were tooth numbers 17, 16, 15, 25, 26 and 27 (Table 1).

**Table 1:** Distribution of the characteristics of study subjects (n=282)

Characteristics	N (%)
Age groups (in years)	
≤ 30	37(13.1)
31-50	147(52.1)
> 50	98(34.8)
Gender	
Male	124(44)
Female	158(56)
Site	
Unilateral	171(60.6)
Bilateral	111(39.4)
Tooth number	
#17	78(27.7)
#16	131(46.5)
#15	97(34.4)
#25	107(37.9)
#26	126(44.7)
#27	80(28.4)

The comparison of the mean values of change in sinus in each of the six teeth across the three age groups showed a statistically significant difference in tooth numbers 17, 26, and 27. In tooth number 17, the highest mean value change occurred in subjects 30 years old or younger when compared with subjects who were between 31 and 50 years old and subjects above 50 years of age (p<0.0001). A similar pattern was observed in tooth number 26, where the mean value of change in the sinus floor position was also significantly higher in subjects 30 years old or younger when compared with subjects who were between 31 and 50 years old and above 50 years of age (p<0.0001).

The mean value of change in the sinus floor position in tooth number 27 was significantly higher in subjects who were between the ages of 31 and 50 years old when compared with those of the other two age groups (p=0.029). Additionally, there was no significant difference between the mean values of subjects less than 30 and more than 50 years old. No statistically significant differences in the mean values of sinus floor position change for the other three teeth (16, 15, and 25) were observed across the three age groups (Table 2).

**Table 2:** Comparison of the mean values of change in sinus in each tooth in relation to age groups *Statistically significant

Tooth Number	Age groups			F-value	p-value
	<=30	31-50	>50		
#17	16.70(10.6)	9.01(4.1)	6.44(3.9)	10.893	<0.0001*
#16	11.72(6.6)	10.04(5.7)	8.10(5.1)	2.978	0.054
#15	9.70(6.2)	10.01(4.8)	7.77(4.6)	2.458	0.091
#25	5.60(2.5)	9.36(4.2)	7.93(3.8)	2.779	0.067
#26	14.61(9.9)	9.47(5.4)	7.39(4.8)	8.595	<0.0001*
#27	6.75(3.0)	8.08(4.4)	5.76(2.9)	3.692	0.029*

The comparison of the mean values of change in sinus floor position in each of the six teeth in relation to gender showed no statistically significant difference (Table 3).

**Table 3:** Comparison of the mean values of change in sinus in each tooth in relation to gender

Tooth Number	Gender		t-value	p-value
	Male	Female		
#17	8.03(5.9)	8.07(4.1)	-0.036	0.971
#16	10.02(6.9)	9.31(4.8)	0.692	0.490
#15	8.27(5.3)	9.61(4.6)	-1.308	0.194
#25	7.99(3.9)	9.04(4.2)	-1.299	0.197
#26	8.74(6.7)	9.54(5.7)	-0.717	0.475
#27	6.46(4.3)	7.40(3.5)	-1.068	0.289

The comparison of the mean values of change in sinus floor position in each of the six teeth, in relation to the two categories of site (unilateral and bilateral), showed statistically significant differences in tooth numbers 16, 15, and 26, where the mean values of change in sinus floor position were significantly higher unilaterally than they were bilaterally. No significant differences were found in the other three teeth (17, 25, and 27) (Table 4).

**Table 4:** Comparison of the mean values of change in sinus in each tooth in relation to distribution *Statistically significant

Tooth Number	Distribution		t-value	p-value
	Unilateral	Bilateral		
#17	9.58(6.7)	7.29(3.8)	1.941	0.056
#16	11.27(6.9)	8.42(4.4)	2.871	0.005*
#15	10.91(5.1)	8.27(4.6)	2.547	0.012*
#25	9.47(3.6)	8.23(4.3)	1.488	0.140
#26	11.70(7.4)	7.47(4.5)	3.984	<0.0001*
#27	7.20(3.4)	6.92(4.1)	0.299	0.766

The comparison of mean values of change in sinus floor position in relation to the molar type (first molar, premolar, and second molar) showed a statistically significant increase in value in the first molar and premolar as compared with the second molar (p=0.002). Multiple comparison tests indicated there was no difference between the first molar and premolar mean values (Table 5).

**Table 5:** Comparison of the mean values of change in sinus in relation to molar type *Statistically significant

Molar type	Mean (Sd)	F-value	p-value
First molar	9.39 (5.9)	6.547	0.002*
Premolar	8.86 (4.5)		
Second molar	7.52 (4.5)		

The molar side (right and left) did not have any effect on the mean values of change in the sinus floor position, as there was no statistically significant difference in the mean values (Table 6).

**Table 6:** Comparison of the mean values of change in sinus in relation to molar side

Molar side	Mean (Sd)	t-value	p-value
Right side	9.05(5.3)	1.473	0.141
Left side	8.44(5.1)		

## Discussion

The results of this study suggest that maxillary sinus pneumatization may occur after posterior tooth extraction. Digital panoramic radiographs were used to assess the change in maxillary sinus floor position following tooth extractions. Radiographs of patients who had a tooth extraction of either the second premolar, the first molar, or the second molar were included in the study. Three reference lines were marked on all radiographs under standard conditions: one line joining the most inferior points of both orbital margins, a vertical line passing through the orbit, bisecting it into two equal halves and a third line running horizontally, parallel to the inferior border of the sinus. Measurements taken included the distance between the maxillary sinus floor to either the second premolar, first molar, or second molar, as these are the teeth whose roots are closest to the sinus and crest of the alveolar ridge. The data were analyzed using SPSS software. Out of 282 study subjects, 56% were females and 52.1% were between the ages of 31 and 50 years old. Site distribution was unilateral in 60.6% and bilateral in 39.4% of subjects. The six teeth that were assessed in this study were teeth numbers 17, 16, 15, 25, 26, and 27.

In the present study, the mean values of change in the sinus floor position in each of the six teeth across the three age groups showed a statistically significant difference in tooth numbers 17, 26, and 27. These differences were clinically appreciable. These values are in agreement with the previous study by Sharan and Madjar [8], showing an increase of 1.83±6 2.46 mm in the sinus dimension for the same site pre and post-extraction without crestal bone preservation. Wagner and colleagues used 400 CT scans to examine the role of sinus pneumatization and residual ridge resorption in maxillary bone loss. They concluded that long-lasting edentulism in the maxillary molar area contributes to mild pneumatization in the walls of the sinus, although the depth of the sinus is independent of dentition as the anatomical variation [16].

Ariji and colleagues assessed normal maxillary sinus volume on axial CT scans in relation to the presence of premolar and molar teeth. For patients over 20 years of age, the volume ranged from 4.56 to 35.21 cm (mean 14.7166.33 cm). Patients with and without maxillary premolars and molars had no significant difference [17].

The comparison of the mean values of change in sinus floor position in relation to the molar type (first molar, premolar, and second molar) showed a statistically significant increase in value in the first molar and premolar compared with the second molar, contrary to the result of Arbel Sharan and Madjar, which showed a larger sinus pneumatization after the loss of a second molar [8].

In regard to the two categories of sites (unilateral and bilateral), results showed a statistically significant difference for teeth numbers 16, 15, and 26, where the mean values of change in sinus floor position were significantly higher on the unilateral site as compared to those of the bilateral site. The data did not provide any significant difference for the other three teeth (17, 25, and 27). According to the present study, gender and molar side groups were not found to be statistically correlated with sinus pneumatization after extraction.

The limitations of this study include a lack of pre-extraction records such as time elapsed since the extraction and radiographs, in addition to the amount of bone loss during the extraction.

## Conclusions

Within the limitations of this study, it can be suggested that tooth extractions in the posterior maxilla may lead to sinus pneumatization and crestal bone loss. If dental implant placement is planned in these cases, the clinician should consider preserving as much bone height as possible by immediate implantation and/or by immediate bone grafting.

## References

[1] Lee KJ (2003). Essentials of Otolaryngology and Head and Neck Surgery, 8th edition.

[2] Misch CE (2015). Maxillary posterior edentulism: treatment options for fixed prostheses. Dental Implant Prosthetics (2nd Edition).

[3] Standring S (2016). Gray´s Anatomy: The Anatomical Basis of Clinical Practice. http://10.1302/0301-620x.91b7.22719.

[4] Laitman JT (2008). Harnessing the hallowed hollows of the head: the mysterious world of the paranasal sinuses. Anat Rec.

[5] Nimigean V, Nimigean VR, Măru N, Sălăvăstru DI, Bădiţă D, Tuculină MJ (2008). The maxillary sinus floor in the oral implantology. Rom J Morphol Embryol.

[6] O'Rahilly R, Müller F, Carpenter S, Swenson R (1983). The nose and paranasal sinuses. Basic Human Anatomy: A Regional Study of Human Structure.

[7] Henson B, Edens MA (2018). Anatomy, Head and Neck, Nose Sinuses. https://www.ncbi.nlm.nih.gov/books/NBK513272/.

[8] Sharan A, Madjar D (2008). Maxillary sinus pneumatization following extractions: a radiographic study. Int J Oral Maxillofac Implants.

[9] Lang J (1989). Clinical Anatomy of the Nose, Nasal Cavity and Paranasal Sinuses. Translated by P. M. Stell. Thieme.

[10] Mauri M, de Oliveira CO, Franche G (2000). Pneumosinus dilatans of the maxillary sinus: case report. Ann Otol Rhino Laryngol.

[11] Tovi F, Gatot A, Fliss DM (1991). Air cyst of the maxillary sinus (Pneumosinus dilatans, pneumocoele). J Laryngol Otol.

[12] Lawson W, Patel ZM, Lin FY (2008). The development and pathologic processes that influence maxillary sinus pneumatization. Anat Rec.

[13] Wang RG, Jiang SC, Gu R (1994). The cartilaginous nasal capsule and embryonic development of human paranasal sinuses. J Otolaryngol.

[14] Misch CE (2008). Contemporary Implant Dentistry. https://books.google.co.in/books?hl=en&lr=&id=x-nv3oQZQ8IC&oi=fnd&pg=PP1&dq=Contemporary+Implant+Dentistry&ots=zXTpDrlP3W&sig=GkoTM4QmnLEe_rSEIxGMMj6lmFc&redir_esc=y#v=onepage&q=Contemporary%20Implant%20Dentistry&f=false.

[15] Levi I, Halperin-Sternfeld M, Horwitz J, Zigdon-Giladi H, Machtei EE (2017). Dimensional changes of the maxillary sinus following tooth extraction in the posterior maxilla with and without socket preservation. Clin Implant Dent Relat Res.

[16] Wagner F, Dvorak G, Nemec S, Pietschmann P, Figl M, Seemann R (2017). A principal components analysis: how pneumatization and edentulism contribute to maxillary atrophy. Oral Dis.

[17] Ariji Y, Ariji E, Yoshiura K, Kanda S (1996). Computed tomographic indices for maxillary sinus size in comparison with the sinus volume. Dento Maxillo Facial Radiology.

